# Noninvasive Detection, Tracking, and Characterization of Aerogel Implants Using Diagnostic Ultrasound

**DOI:** 10.3390/polym14040722

**Published:** 2022-02-13

**Authors:** Sagar Ghimire, Martina Rodriguez Sala, Swetha Chandrasekaran, Grigorios Raptopoulos, Marcus Worsley, Patrina Paraskevopoulou, Nicholas Leventis, Firouzeh Sabri

**Affiliations:** 1 Department of Physics and Material Science, The University of Memphis, Memphis, TN 38152, USA; sghmire1@memphis.edu (S.G.); mrdrgzsl@memphis.edu (M.R.S.); 2 Lawrence Livermore National Laboratory, Livermore, CA 94551, USA; chandrasekar2@llnl.gov (S.C.); worsley1@llnl.gov (M.W.); 3 Department of Chemistry, National and Kapodistrian University of Athens, 15771 Athens, Greece; grigorisrap@chem.uoa.gr (G.R.); paraskevopoulou@chem.uoa.gr (P.P.); 4 Department of Chemistry, Missouri University of Science and Technology, Rolla, MO 65409, USA; nleventis@aerogel.com

**Keywords:** aerogel, B-mode, acoustic attenuation, ultrasound

## Abstract

Medical implants are routinely tracked and monitored using different techniques, such as MRI, X-ray, and ultrasound. Due to the need for ionizing radiation, the two former methods pose a significant risk to tissue. Ultrasound imaging, however, is non-invasive and presents no known risk to human tissue. Aerogels are an emerging material with great potential in biomedical implants. While qualitative observation of ultrasound images by experts can already provide a lot of information about the implants and the surrounding structures, this paper describes the development and study of two simple B-Mode image analysis techniques based on attenuation measurements and echogenicity comparisons, which can further enhance the study of the biological tissues and implants, especially of different types of biocompatible aerogels.

## 1. Introduction

Non-invasive diagnostic imaging techniques are needed to track biomedical implants, evaluate their efficacy, and monitor any adverse reactions [1,2]. Routinely used imaging techniques include magnetic resonance imaging (MRI), computed tomography (CT), X-ray imaging, positron emission tomography (PET), and ultrasound (US) imaging [1]. Diagnostic US imaging is a desirable technique since it is noninvasive, portable, and more importantly, does not rely on ionizing radiation for image formation [3]. Concerns regarding acoustic cavitation have been raised [4]. However, this is not a concern for the low-level exposure that is needed for imaging purposes; additionally, the diagnostic frequency range that is routinely used is considered very low risk [5].

Diagnostic US devices typically operate in the frequency range of 2–18 MHz and wave generation occurs by means of electromechanical transducers using piezoelectric materials [6]. Sound waves are reflected to different degrees at a boundary between two media that have different acoustic impedances and image formation is primarily driven by the physical parameters of the different materials [7,8]. Different modes of US are used in medical settings. These include A-mode, B-mode, and M-mode [7]. B-mode ultrasonography is a particularly valuable tool for muscle evaluation, despite being a complex process, which involves beamforming, envelope detection, and Log compression [9,10]. Ultrasonography has been used for evaluating hard and soft tissue as well as implant stability.

The potential of aerogels as biomaterials for biomedical implants has now been firmly established and has been the subject of many recent studies [11]. Aerogels have shown great promise for applications, such as drug delivery [12,13], neural prosthetics [14,15,16,17,18,19], and cardiovascular [20,21] and bone implants [22,23,24]. Of particular interest is in vitro [18,19,25,26,27,28] and in vivo [15,16,17] studies that evaluate the response of neurons to aerogels. Studies have shown that aerogels accelerate the regeneration rate of neurons and are identifiable using traditional imaging techniques, in vivo.

Here, we present a comprehensive study of the acoustic response of different types of aerogels, tested under biologically relevant conditions. The aerogels that were chosen for this study were carefully selected to cover a broad range of pore diameters (nm-μm) and Young’s moduli (Pa-MPa) and were imaged using a clinical US device (B-mode) while being inserted subcutaneously (SC) and sub-muscularly (SM) as well as in an aqueous environment (Aq). The effect of the wave frequency and ambient temperature on image formation and resolution was also investigated. The degree of attenuation by each aerogel type, acoustic impedance, and the speed of sound in each case was calculated and quantified. This study paves the way for future studies utilizing aerogels for biomedical applications.

## 2. Materials and Methods

### 2.1. Aerogel Selection and Sample Preparation

Aerogels were chosen for this study based on their pore diameter (φ) range and Young’s modulus (Y) values. Aerogel types investigated in this study are briefly described below and summarized in Table 1. These include (1) polyurea-crosslinked silica aerogel (X-silica) [14,15,16,17,18,19,29,30], (2) X-silica-La_2_O_2_S:Eu composites, (3) two forms of superelastic shape-memory polyurethane aerogels (SMPU): referred to as Mix-14 and Mix-18 [31], (4) two forms of carbon aerogels (CA): acetic acid-catalyzed resorcinol formaldehyde aerogels (ARF-CA) and base-catalyzed resorcinol formaldehyde aerogels (BRF-CA) [32,33], and (5) two forms of polyurea-crosslinked calcium alginate (X-Ca-Alg-1 and X-Ca-Alg-2) aerogels [34,35]. All aerogels were synthesized separately as monoliths and after completing the drying process each monolith was carefully segmented into 1.0 cm × 0.8 cm × 0.4 cm sample sizes using a sharp blade. Detailed synthesis protocols for each aerogel type can be found in previously published literature as shown in Table 1 and are briefly summarized below.

X-silica aerogels: Prepared based on silica formed from a colloidal suspension in methanol, deionized water, tetramethyl orthosilicate and (3-aminopropyl) triethoxysilane. This suspension was first allowed to gel and wet gels were crosslinked at the nanoscopic level with a triisocyanate (Desmodur N3200 from Covestro, Leverkusen, DE) and dried using a CO_2_ supercritical drying stage [14,15,16,17,18,19].

X-silica-phosphor composites: These aerogels were formed by incorporating 10 weight percentage of lanthanum oxysulfide (La_2_O_2_S:Eu) (Phosphor Technology, Hertfordshire, UK, lot number 23010 SKL63/F-X) powder in the colloidal suspension mentioned earlier in the X-silica aerogels methods section (gelation stage). The composites were CPD with the same protocol as the non-composite counterparts [36,37].

Shape-memory polyurethane aerogels (SMPU): Two different types of SMPU aerogels Mix-14 and Mix-18, were prepared using a triisocyanate incorporating three flexible/aliphatic branches around a rigid isocyanurate core (Desmodour N3300A from Covestro) and different amounts of three short diols, derivatives of ethylene glycol: diethylene glycol (DEG), triethylene glycol (TEG), and tetraethylene glycol (TTEG), hence the prefix “Mix-“ in the sample names [31,38,39].

Carbon aerogels: These aerogels were prepared by combining different proportions of water, acetic acid, resorcinol, and formaldehyde to form gels. The gels were then cured, bathed in acetone, and finally dried super-critically. Finally, the aerogels were heated at 1050 °C for 3 h. Given that ARF-CA and BRF-CA contain different proportions of the precursor chemicals, their final Y and φ values differ substantially and were valuable for our study [32,33].

Polyurea-crosslinked calcium alginate aerogels (X-Ca-Alginate): Two types of X-Ca-Alginate aerogels, X-Ca-Alg-1 and X-Ca-Alg-2, were prepared from Ca-alginate hydrogels, which in turn were the result of the reaction of CaCO_3_, δ-gluconolactone (GDL) and an Aq sodium alginate (PROTANAL LF 240 D; G/M = 0.43–0.54) solution. Ca-alginate hydrogels were washed with acetonitrile and were reacted (crosslinked in the same sense described for X-silica aerogels) with Desmodur N3300 (the EU version of Desmodur N3300A described above) [34,35]. X-Ca-Alg-1 and X-Ca-Alg-2 differed in the initial concentrations of Aq sodium alginate (0.9 and 1.8% *w*/*w*, respectively), while the concentration of Desmodur N3300 in the crosslinking bath was the same in both cases (26.8% *w*/*w*). That resulted in different polyurea contents (93 and 59% *w*/*w*, respectively).

### 2.2. Evaluation of Physical Properties

Young’s Modulus (Y): The compressive modulus for each aerogel type was measured using a motorized test Stand ESM303 (Mark-10, Copiague, NY, USA) equipped with a Series 5 (Mark-10) force gauge, set to deliver a compression rate of 15 mm/min. Using the “travel” and “load” data and Equation (1) in Table 2, the Y value was calculated.

Bulk density (ρ): The bulk density of each aerogel sample was calculated from the mass of the sample (Fisher EMD 100A microbalance, Waltham, MA, USA) and the physical dimensions of the sample.

Pore Diameter (φ): The reported pore diameters were measured using a S-4700 scanning electron microscope (Hitachi, Santa Clara, CA, USA). Images were exported to ImageJ (version 1.53a) where the scale bar was used for length calibration. The straight-line tool was used to quantify the pore diameters for each aerogel type. In each case, three independent regions were imaged (*N*_1_ = 3), and each image contained a minimum of one hundred measurements (*N*_2_ = 100).

Attenuation coefficient (α): The attenuation coefficient was measured using the US image analysis method explained in Section 2.4. α value for each aerogel type was measured three times (*N* = 3) for each scan frequency.

Acoustic Impedance (Z): To calculate the acoustic impedance of each aerogel, previously measured ρ and Y values were inserted into Equation (2), Table 2.

Speed (v): The speed of sound (v) in solid is influenced by Young’s modulus and bulk density of the material. The aforementioned parameters Y and ρ were used to calculate the speed of sound in each aerogel type using Equation (2b), Table 2.

### 2.3. Experimental Setup for Ultrasound Image Acquisition

A medical diagnostic US system Edan U50 Prime (Edan, San Diego, CA, USA) coupled with two linear array transducers were used in this study. Probes included an L742UB (radius: 60 mm, elements: 128, Bandwidth: 5–10 MHz, center frequency: 7.5 MHz, scanning depth: 30–130 mm) and L1042UB (radius: 38 mm, elements: 128, Bandwidth: 8–12 MHz, center frequency: 9.5 MHz, scanning depth: 20–110 mm) (Edan, San Diego, CA, USA) recommended for superficial, vascular, and musculoskeletal scans. Aquasonic clear US gel (Parker Laboratories. Inc., Fairfield, NJ, USA) was used as a coupling agent between the transducer and tissue. For each trial, a 0.5 cm thick gel layer was maintained between transducer and tissue.

**Figure 1:**
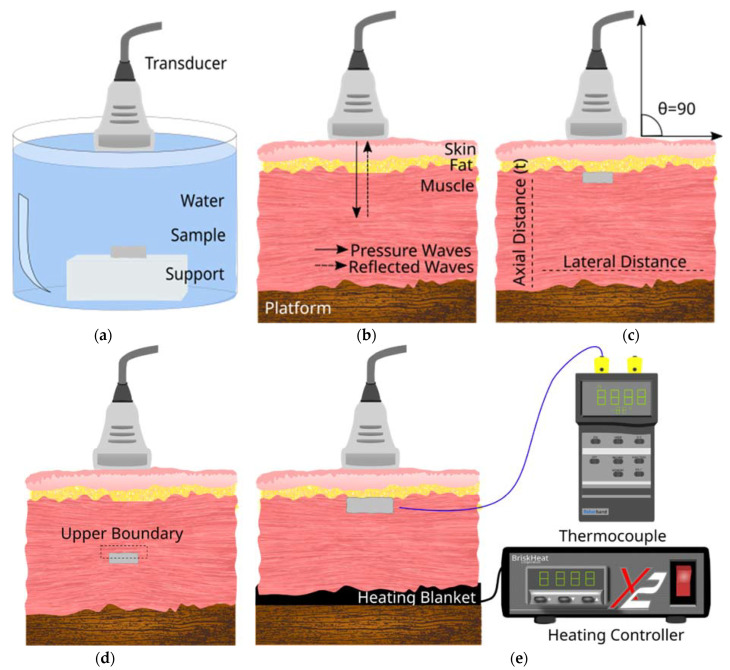
Schematic diagram of the cross-section view of the different configurations used for diagnostic US imaging of aerogel. (**a**) Aerogel samples were tested in an Aq environment by placing them on top of sample support. (**b**) Configuration used to image the tissue without any implants. This served as the baseline for image analysis. Continuous arrows show pressure waves propagating and though the tissue and implant. Discontinuous arrows show reflected pressure waves from the Z difference within the tissue and implant area. (**c**) Configuration where implant insertion is SC (**d**) Configuration where implant insertion is SM. (**e**) Configuration with temperature controlling heating blanket and thermocouple for US imaging at different temperatures. The angle of scan was maintained at 90° that corresponds to the 0° in US device settings.

To simulate different in vivo conditions, several experimental configurations were tested and are shown in Figure 1, where a schematic diagram of the cross-sectional view of the different configurations is shown. The relative position of the ultrasound probe, sample, and tissue layers have been indicated. The arrows indicate the direction of travel of the pressure wave (incident and reflected). First, to establish a baseline, samples were imaged in an Aq environment in the absence of any tissue layers (Figure 1a). A suitable support platform was identified and for the sake of consistency, the same platform configuration was used for all tests. Once the sample was secured inside an Aq bath of a known volume and temperature, the transducer was secured at 1 cm from the sample, positioned directly above it. To mimic in vivo conditions, grocery store-grade tissue of dimensions 12 cm × 7 cm × 2.44 cm (devoid of any skeletal structure and containing minimum fat content) was acquired and used as the tissue layer. The tissue consisted of muscle, fat, and skin layer and was used as a combined layer structure to evaluate the degree of attenuation caused by each layer and in combination. To establish a second baseline, the tissue alone was imaged (Figure 1b) and these measurements served as the baseline for image analysis and calculations explained in later sections. No aerogels were imaged at this stage.

In the presence of aerogels, ultrasound images were collected both subcutaneously (SC) (Figure 1c) and sub-muscularly (SM) (Figure 1d). SC implants were placed at a depth of 0.1 cm while SM samples were placed at a depth of 0.7 cm. In both cases, to place the aerogel, a lateral incision was first made with minimum disturbance to the layer structure. In all cases, the transducer was fixed vertically above the tissue layer (scan angle 0°) corresponding to an angle of 90° between the transducer and tissue (indicated on the schematic diagram). The transducer was then moved horizontally across the entire sample area and images were collected in 2 mm intervals (N = 5) for the following frequencies: 6.5, 7.5, 8, 8.5, 9.5, 11, 13, and 13.4 MHz.

To more accurately mimic in vivo conditions, the above experiment was repeated at body temperature (37 °C) by first warming up the tissue to 37 °C and acquiring US images at this temperature. To maintain a steady temperature, a UNV161001 heating pad (Briskheat, Columbus, OH, USA) equipped with a temperature control unit X2-220JT (Brisk-heat) was employed (Figure 1e). For all configurations and measurements, the following device settings were used: Dynamic range of 98 dB, mechanical index below 1.0, gain of 50 dB, depth of field 3.9 cm, and time gain compensation (TGC) was turned off at the lower depth to reduce the attenuation compensation for both transducers. Images were saved as BMP files and used for quantitative image analysis as described in Section 2.4.

### 2.4. Image Processing and Analysis

**Figure 2:**
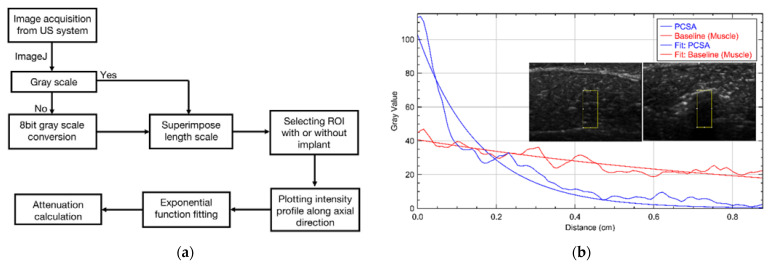
(**a**) Flowchart showing the attenuation analysis procedure used in this study. US images were exported to ImageJ (NIH open-source software) to scale the measurements, selecting the region of interest (ROI) and exponentially fitting the intensity profile at 6.5 MHz frequency. (**b**) Representation of exponential fit of selected ROI of two different B-mode images of baseline (left) and PCSA (right) at 6.5 MHz frequency.

ImageJ (version 1.53a) software was used for all image analysis and calibrated such that the length scale corresponded to the scale of the US images. Grayscale US images were exported from the Edan U50 unit and all image processing (color mapping, intensity mapping, etc.) was completed in ImageJ. The flowchart in Figure 2a summarizes the steps taken to arrive at the attenuation coefficient. Figure 2b shows a representative intensity profile where the *x*-axis corresponds to t and the *y* axis represents the intensity along the axial direction.

### 2.5. Attenuation Calculation

Attenuation (α) values were obtained from the intensity profiles measured axially (t) by fitting Equation (3a), Table 2 to this data from images taken at the fundamental frequencies (6.5, 7.5, 8.5, 11 MHz). Aerogels were imaged at all available frequencies. However, to avoid calculation errors, α was calculated only from the images taken at the fundamental frequencies (6.5, 7.5, 8.5, 11 MHz) and not the harmonic frequencies since our calculations rely on pixel intensity. Therefore, for qualitative analysis harmonic images are preferred (because of the various parameters that a US system applies to improve the image quality) while for quantitative analysis, fundamental frequencies are more appropriate.

The method developed in this paper utilizes the idea that highly attenuating structures cast posterior shadows in US images. The pixel intensity at the upper boundary of aerogels and the subsequent decrease in intensity along the wave path is determined by the mechanical properties of each aerogel type and their absorption and scattering properties. An exponential fit using Equation (3a), Table 2, of the pixel intensity profile from the boundary to the posterior shadow determines α with a goodness of fit > 0.8 for most US images. Moreover, the intensity depends upon the frequency used for the scan suggesting the dependence of the attenuation upon the frequency.

### 2.6. Echogenicity

**Figure 3:**
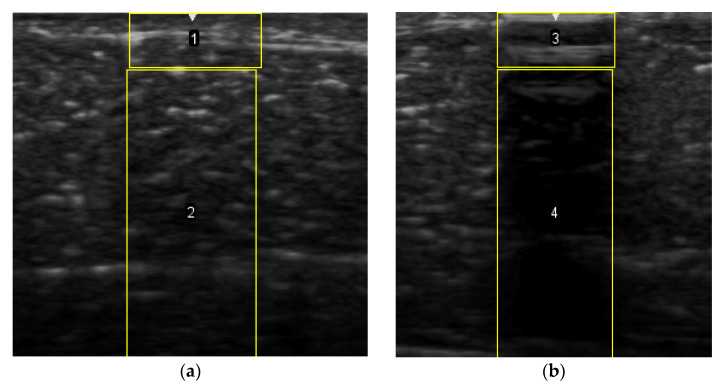
(**a**) Selection of ROI-1 and ROI-2 in the B-mode image of the muscle without implants. (**b**) Selection of ROI-3 and ROI-4 in the B-mode image with aerogel implants.

To measure the echogenicity of each aerogel type, two ROIs were strategically selected from each B-mode image and indicated with a clear boundary in Figure 3. These correspond to *top* ROI which represents the implant area and *bottom* ROI representing the posterior shadowing region. Figure 3a shows these two regions in the absence of any aerogel implants while Figure 3b captures the same regions in the presence of an implant (ROI-3 and ROI-4). In each case, the Mean Pixel Intensity (MPI) of the two ROIs was measured using the measure function in ImageJ. MPI of ROI-1 and ROI-2 formed the baseline for the percentage change calculation, which was arrived at using Equation (4a,b)—Table 2. Based on the value of the ROI, aerogels were classified as hyperechoic, isoechoic, hypoechoic, and anechoic and summarized in Table 3 where ΔE can vary between −100% and +255%. Acoustic impedance mismatch between the aerogels and tissue was calculated using a percentage difference method, indicated in Table 2 by Equation (6).

To calculate the standard error of the mean, the MPI of each ROI was collected from three (N = 3) different images for the same frequency and for the sake of consistency all the same device settings were used.

## 3. Results

### 3.1. Aerogel Characterisation Results

The physical properties of aerogels used in this investigation have been summarized in Table 4. Measurements indicate that X-silica and X-silica-La_2_O_2_S:Eu aerogels have the highest Youngs‘ modulus (8.35 and 11.4 MPa, respectively) which is an expected outcome. X-Ca-alginate and CA aerogels have Y values of a similar range (0.91–1.29 MPa) and SMPU Mix-14 and Mix-18 are the most flexible aerogels (0.3–0.4 Mpa) among the variety studied here. Aerogel bulk densities fell in the range of 650–950 kg/m^3^ except for X-Ca-alginate aerogels which were in the range of 88–150 kg/m^3^. The calculated value for the speed of sound (v) was greatest in X-silica and X-silica-La_2_O_2_S:Eu (80.58–90.21 m/s). CA and SMPU aerogels had the lowest values (25–35 m/s) with X-Ca-alginate aerogels in between. Overall, the values for v in these aerogels are very low but not surprising. Previous studies have shown that propagation of sound in aerogels occurs at very low speeds when compared to other solids [43]. Z values were found to be higher in X-silica and X-silica-La_2_O_2_S:Eu (3–3.5 Mrayl) compared to the rest of the aerogels studied (0.34–0.95 Mrayl). Φ had a range between 40 nm and 5 µm with BRF-CA, X-Ca-alginate, and X-silica having pore diameters on the nanometer scale while ARF-CA and SMPU had micrometer sized pore diameters.

### 3.2. Correlation between Acoustic Impedance (Z), Young’s Modulus, (Y) and Speed (v)

**Figure 4:**
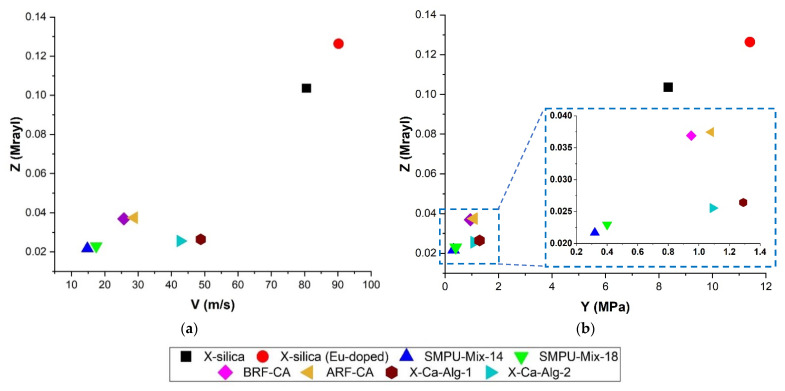
(**a**) Correlation between impedance values and sound speed for each type of aerogel used in this study. (**b**) Impedance as a function of Young’s modulus for all the aerogels used in the experiment. Both (**a**,**b**) represent data in an Aq environment.

Figure 4 shows the relationship between Z vs. v (Figure 4a) and Z vs. Y (Figure 4b) for the different types of aerogels studied here. In both cases, a positive slope can be seen. Speed of propagation is highest in aerogels with the greatest Z values (X-silica-La_2_O_2_S:Eu 0.126 Mrayl, and X-silica 0.104 Mrayl) (Figure 4a). The speed of propagation drops significantly for aerogels with lower Z values, with a noticeable reduction between the silica-based aerogels (102.9 m/s and 84.26 m/s, respectively) and the alginate-based aerogels with v values in the range of 101.41 m/s and 121.77 m/s. As a first approximation, Equation (2a)—Table 2, suggests a linear relationship between Z and v for homogenous materials. While results presented in Figure 4a deviate slightly from a linear behavior, the overall trend is consistent with theoretical expectations [40]. A linear trend with a positive slope can be seen in Figure 4b where Z increases with Y.

### 3.3. B-Mode Images

Table 5 (part 1 and 2) show representative US images at 6.5 MHz of the different aerogel types while embedded, in three different formats: grayscale (Left), normalized intensity maps (Center), and 3D intensity map where color represents the pixel intensity (Right). In the 3D normalized intensity map (Table 5, Right), the *x*-axis represents the lateral resolution of the corresponding B-mode image, the *y*-axis, the axial resolution, and the *z*-axis, the pixel intensity value of the image which makes the visualization of the implant region easier. The normalized 2D intensity map (Table 5, Center) provides a detailed image of the structure, and it complements the 3D view of the color graph (Right). The grayscale image (Left) is the original ultrasound image collected by the Edan U50 device. The “Muscle” images represent the baseline for all three image forms and can be seen that it is relatively homogeneous with no remarkable features.

After insertion of aerogel implants (Rows 2 and beyond), considerable changes are seen in all three image forms. The 3D intensity plot (Right column) clearly identifies the 3D profile of the aerogel implant providing us with depth information. The upper boundary of the aerogel implant can be easily identified with an intense red streak for some of the aerogels studied here (Rows 2, 3, 5, 6, 8, 9). This arises from an impedance mismatch between the aerogel and its immediate environment. It is distinguishable from the tissue-transducer boundary which can also be identified with a separate red “streak”, marked on the control image, Row 1, indicated with white and black arrows. Strong posterior shadowing is observed in all aerogels (Rows 4, 5, 8, 9) except in BRF-CA and ARF-CA which show a waterfall appearance (Table 5, part 2). X-silica and Eu-X-silica also have distinct linear boundaries, whereas SMPU and alginate-based aerogels have an irregular boundary, and for CA aerogels, the boundary is not very distinct, which will be discussed in later sections.

### 3.4. Attenuation Coefficient (α) of Aerogel Implants

The impact of scan frequency, environment (Aq, SC, SM), and temperature on the attenuation coefficient (α) of aerogels were thoroughly investigated and reported here. The relationship between α, Y, and φ was also explored and discussed in subsequent sections.

#### 3.4.1. Effect of Environments on Attenuation Coefficient (α)

Table 6 summarizes the attenuation coefficient values that were calculated from the B-Mode images in the three environments: Aq, SC, and SM. The data provided in Table 6 represents a scan frequency of 8.5 MHz and reflects the trends seen at all other frequencies between 6.5–11 MHz. To avoid redundancy, only results from one frequency are shown.

Attenuation measured from the proposed method shows that the degree of attenuation is greatest when aerogels were inserted SC and lowest when placed in an Aq environment. This trend was observed for all aerogel types, though the amount of attenuation varied for different aerogels. X-silica-La_2_O_2_S:Eu showed the highest amount of attenuation with values of 8.21 dB/cm, 13.76 dB/cm, and 20.84 dB/cm at 8.5 MHz for the different environments. This corresponds to a 1.5-fold increase in SC over Aq and a 0.67-fold decrease in SM. The overall amount of attenuation was the lowest in the CA aerogels when compared to the other aerogel types. Among the CA aerogel variety, the least amount of attenuation was observed in AFR-CA aerogel where α was 2.39 dB/cm, 3.27 dB/cm, 4.77 dB/cm in Aq, SM, and SC, respectively. α increased over 0.3-fold in SC compared to Aq and with no change in SM. The same trends were observed for all other frequencies.

#### 3.4.2. Attenuation Coefficient (α) Dependency on Scan Frequency

**Figure 5:**
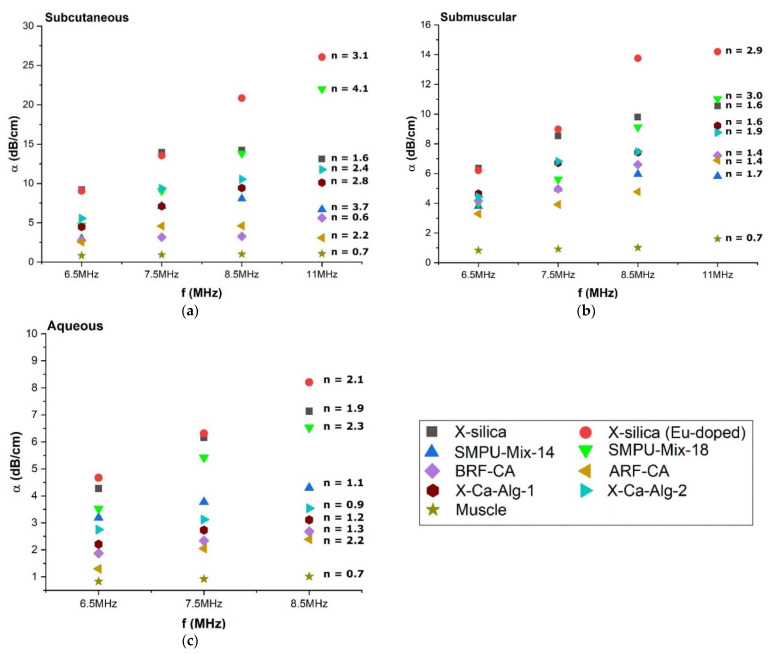
Attenuation versus frequency plot for three different environments, (**a**) Subcutaneous (**b**) Submuscular and (**c**) Aqueous. The attenuation coefficient dependence can be seen from the graphs above, α = α_ο_f^n^.

The degree of attenuation was dependent on the scan frequency and confirms the theoretical understanding that is suggested by Equation (3b), Table 2. The attenuation coefficient, α, was calculated for each aerogel type, in three different environments (SC, SM, and Aq) and at frequencies; 6.5, 7.5, 8.5, and 11 MHz summarized in Figure 5. From Equation (3b), Table 2, we can also see that the attenuation is dependent upon the nth power of the frequency. Power law fitting of α vs. f was conducted for each measurement and the value of *n* was calculated for each aerogel type, at each frequency (indicated on the graphs in Figure 5a–c). The *n* value for muscle that we arrived at using this method was *n* = 0.7 and closely matched previously reported values [41] indicating that the method adopted here for extracting *n* values is indeed correct.

#### 3.4.3. Effect of Temperature on Attenuation Coefficient (α)

**Figure 6:**
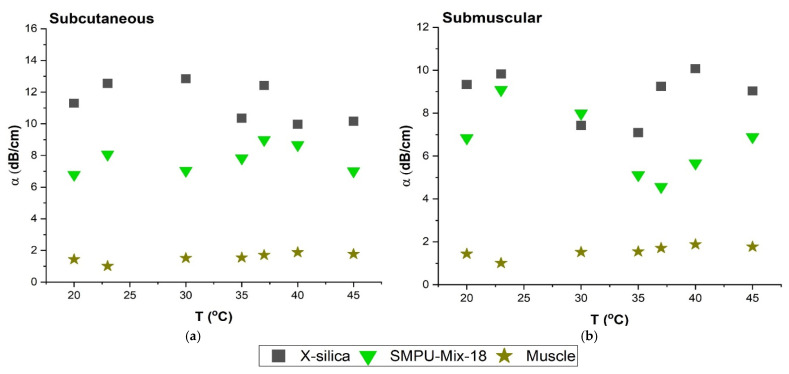
Comparison of attenuation coefficient (α) at temperature range of 20–45 °C at 6.5 MHz. (**a**) α in SC region (**b**) α in SM region. Temperature range is consistent with small fluctuations.

The attenuation measurements were carried out in the temperature range of 20–45 °C in increments of 5 °C for all aerogel types. Figure 6 shows the effect of temperature on the degree of the attenuation for SC and SM configurations for two types of aerogels; X-silica and SMPU-Mix-18 representing the results of this test at a scan frequency of 6.5 MHz. It can be concluded that the degree of attenuation does not have a strong dependency on ambient temperature when tested in the range of 20–45 °C. Temperatures above 45 °C were not investigated since they would not have physiological relevance.

#### 3.4.4. Relationship between Attenuation Coefficient (α), Young’s Modulus (Y), and Pore Diameter (φ)

**Figure 7:**
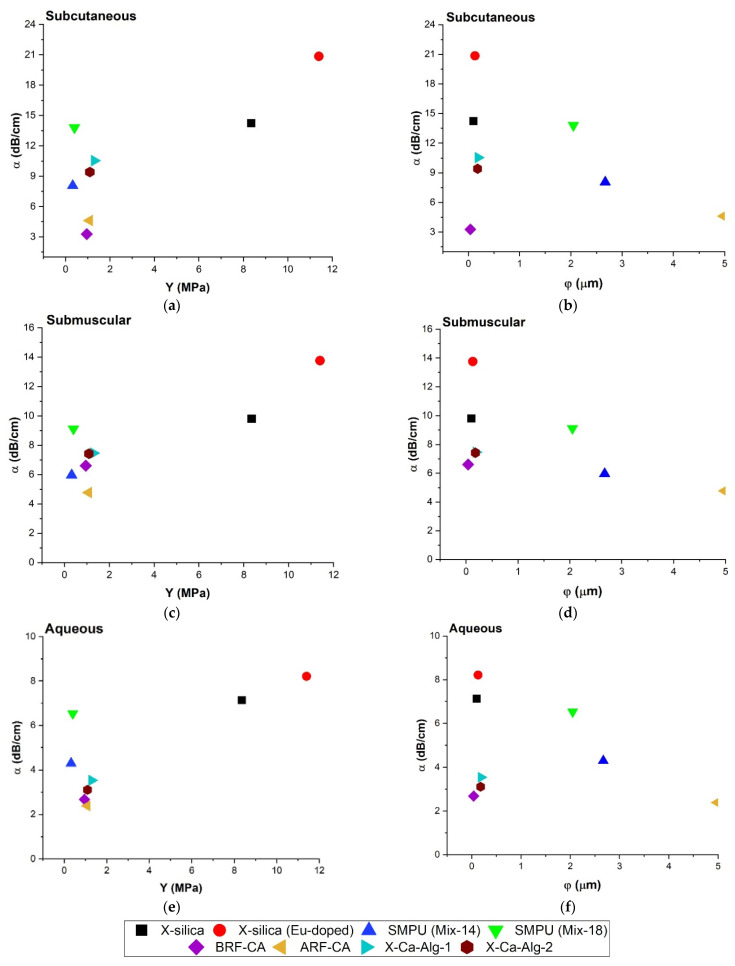
Comparison of attenuation coefficient (α) at 8.5 MHz, Young’s modulus (Y), and Pore diameter (φ) for all three configurations; Aq environment samples inserted SC and SM measured at a scan frequency of 8.5 MHz. (**a**) α vs. Y in SC region, (**b**) α vs. φ in SC region, (**c**) α vs. Y in SM region, and (**d**) α vs. φ in SM region. (**e**,**f**) represent α vs. Y and α vs. φ respectively in an Aq environment.

The effect of Young’s modulus and pore diameter on attenuation was studied and reported here. Figure 7a,c,e show the relationship between Y vs. α (at 8.5 MHz) for SC, SM, and Aq, respectively. Results indicate that samples with higher Y values correspond to a greater degree of attenuation, namely for X-silica-based aerogels. Aerogels with Y values less than 2 MPa, however, did not show a clear trend which suggests that other parameters are influencing the overall behavior. The attenuation coefficient did show dependency on the environment and the most attenuation was observed for the SC configuration. The behavior of α vs. φ is shown in Figure 7b,d,f. (Equation (5a,b)).

Table 2 suggests an inverse square relationship between φ and α. For samples Eu-doped X-silica, SMPU-Mix 14, SMPU-Mix 18, and ARF-CA our results follow theory for all environments. However, for X-silica, X-Ca-Alg-1, X-Ca-Alg-2, and BRF-CA since they have similar pore diameters (0.1–1 μm) they are clustered at one end of the graph and are only distinguished from one another because of differences in their pore density (γ) and potentially their porosity (ϑ). Investigating the direct correlation between porosity and other parameters will be the subject of future studies.

**Figure 8:**
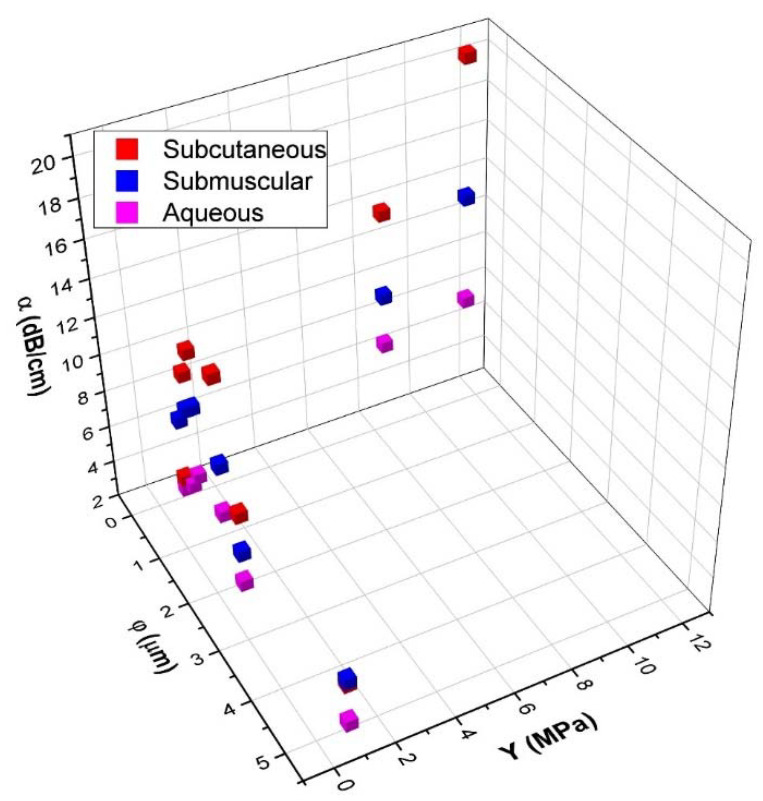
Comparison of attenuation coefficient (α) at 8.5 MHz, Young’s modulus (Y) and pore-diameter (φ) for SC, SM, and Aq implants.

To better understand the relationship between α, Y, and φ, a 3D scatter plot was created from data presented in Figure 7 and is shown in Figure 8. The influence of φ and Y on α can be observed with two clear patterns. With samples of Y below 2 MPa, the influence of φ on α is greater than Y, where α decreases with increasing φ. For samples with φ below 0.5 µm, Y has a higher influence where an increase in Y corresponds to an increase of α.

#### 3.4.5. Acoustic Impedance (Z) and Attenuation Coefficient (α)

**Figure 9:**
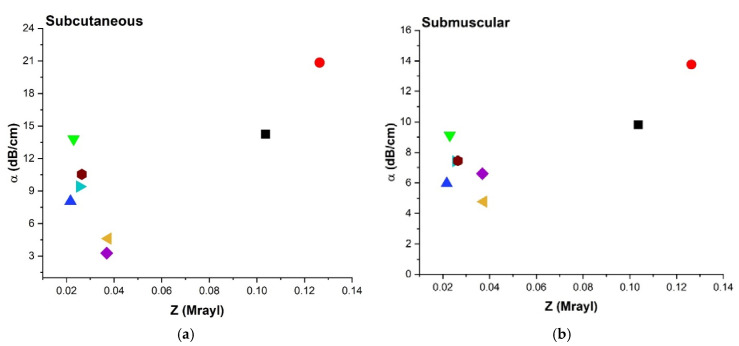

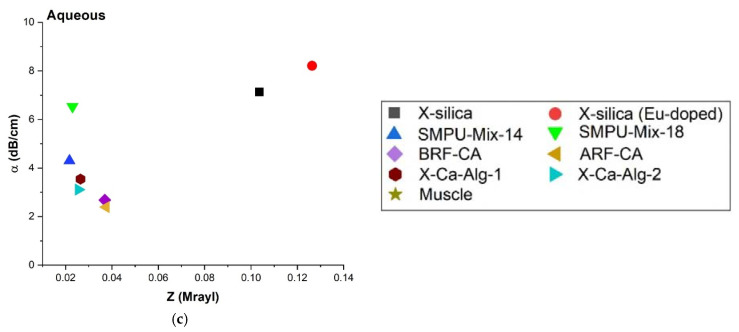
Comparison of attenuation coefficient (α) at 8.5 MHz and acoustic impedance (Z) for all three configurations; (**a**) SC, (**b**) SM, and (**c**) Aq environment.

The relationship between α and Z was investigated and shown in Figure 9 for a representative frequency of 8.5 MHz, and it closely follows the trend that was observed in Figure 7a,c,e for α vs. Y. A similar trend can be attributed to the fact that Z depends on Y^1/2^ as seen in Equation (2a), Table 2.

### 3.5. Echogenicity

**Figure 10:**
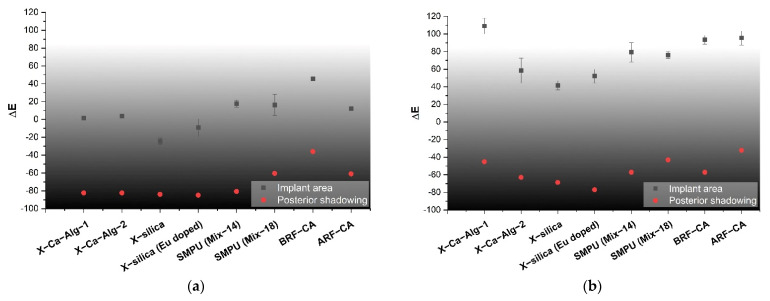
Echogenicity of the aerogels at 8.5 MHz tabulated according to the classifications indicated in Table 3: (**a**) SC and (**b**) SM compared to control, calculated using Equation (4a,b), Table 2.

The quantification of pixel intensity of US images taken at 8.5 MHz was performed for two distinct image areas; (1) implant and (2) the posterior shadowing region and applying Equation (4), Table 2. These regions correspond to ROI 1 and 3 of Figure 3 for implant area and, ROI 2 and 4 for posterior shadowing. By evaluating these two regions (described in Section 2.6) for each US image we were able to calculate the echogenicity of the different types of aerogels. Results indicate a strong dependency of Echogenicity on implant location (SC vs. SM). Figure 10a shows the echogenicity (ΔE) of the aerogels in the SC region while Figure 10b presents the data for the SM region. In the SC case, X-silica is most hypoechoic (ΔE = −24%), while BRF-CA is most hyperechoic (ΔE = +50%). In the SM case, X-silica is the least hyperechoic aerogel (ΔE = +40%) and X-Ca-Alg-1 is the most hyperechoic with a ΔE value of +120%. All the aerogels in the SM region show hyperechogenicity with X-Ca-Alg-1 (109%) being the highest and X-silica (42%) being the least (Figure 10b). By comparing Figure 10a and Figure 10b, it can be seen that the overall echogenicity has increased in the SM region which will enhance the contrast and better identify the aerogel implant. Error bars associated with the Posterior Shadowing were statistically insignificant and hence not shown.

## 4. Discussion

### 4.1. Ultrasonography and Aerogel Dimensional Analyis

In most cases, aerogels resembled “hard tissue”. The horizontal dimension of the aerogel samples inferred from the upper boundary of a US image (Figure 1c) had a mean difference of −0.03–0.07 cm when compared to values calculated from direct observation. This reduction of the lateral dimension did not appear to be frequency dependent for the frequencies studied here.

### 4.2. Wave Propagation in Aerogels

The speed of sound is directly proportional to Y^1/2^ and inversely proportional to the density of the medium (Equation (2b), Table 2). Based on the range of v values that we have reported in this work (14–90 m/s) three groups of aerogels can be formed: (1) Aerogels with large ρ and large Y values (X-silica and Eu-X-silica) which have high v, (2) large ρ and small Y (CA and SMPU aerogels), which have low speed values, and (3) small ρ and large Y (X-Ca-Alg-1 and X-Ca-Alg-2) having comparatively high speed values.

### 4.3. Attenuation Coefficient (α) of Aerogels

The method of calculating α used in our study provides an improvement compared to previous methods [44] because our method does not depend on the uniformity of pixel distribution which can lead to incorrect attenuation values.

#### 4.3.1. Attenuation Coefficient (α) Comparison at Different Frequencies

The attenuation Coefficient (α) is expected to depend on the scan frequency, as indicated in Equation (3), Table 2. As demonstrated, our results confirm this behavior (Figure 5) in the range of 6.5 to 11 MHz which allows us to extrapolate the degree of attenuation at other frequencies.

#### 4.3.2. Effect of Temperature on Attenuation Coefficient (α)

The effect of temperature on the ultrasonography of aerogels was also investigated. Results indicate that between room temperature and 45°C, for a given frequency, the attenuation coefficient, α, does not show a strong dependency on temperature for either SC or SM placement. Figure 6 shows representative values for f = 6.5 MHz and shows the same trend that was observed at other frequencies. These results are consistent with reports from other studies in this temperature range for the attenuation calculation of the tissues [45,46].

#### 4.3.3. Attenuation Coefficient (α), Pore Diameter (φ) and Young’s Modulus (Y)

Figure 7b,d,f show a decrease in α with an increase in φ. Doped X-silica, X-Ca-Alg-1, SMPU-Mix-14 and ARF-CA have a larger difference in the pore diameter compared with each other and show a decrease in attenuation which is in agreement with Equation (5a,b), Table 2 where α is inversely proportional to φ^2^. Figure 7a,c,d show the direct relationship between α and Y. In the range of 0–2 MPa, a greater degree of scattering α was observed when compared to Y > 2 MPa behavior.

To better understand the correlation between the different parameters, it was important to identify the relationship between the attenuation and Young’s modulus which is presented below:

The Speed of sound in a solid medium is given by Equation (2b), Table 2. We also know that the speed is defined by wavelength and frequency as follows:

v = λ·f(7)

Equating these two equations indicates that:

v = √Y/ρ = λ·f(8)

The dependence of the frequency in attenuation is given by Equation (3b), Table 2, which can be rewritten as:

f = (α/α_o_)^1/n^ (9)

Substituting Equation (9) into Equation (8), we arrive at:

√Y/ρ = λ·(α/α_o_)^1/n^ (10)

Rearranging the above equation leads us to:

α = (α_o_/λ^n^)·(Y/ρ)^n/2^ (11)

At a given frequency, when *n* = 2, α becomes linearly proportional to Y/ρ. Few of the aerogels tested in our study have a value of *n* close to 2. A perfect linear fit, therefore, cannot be seen in Figure 7 because of this.

Figure 8 shows a comparison between α, Y and φ where the influence of φ and Y to α can be observed with two clear patterns. With samples of Y below 2 MPa, the influence of φ on α is greater than Y, where α decreases with increasing φ. On samples with φ below 0.5 µm, Y has a higher influence where an increase in Y corresponds to an increase of α.

#### 4.3.4. Attenuation Coefficient (α), Impedance

Figure 9a–c shows α plotted against Z showing a similar trend to that of α vs. Y (Figure 7a,c,d). This similarity can be explained by Equation (2a), Table 2.

### 4.4. Echogenicity

Previous studies have shown that the impedance of tissue is of the order of 1.6 MRayl [47]. Using this value, the acoustic impedance mismatch was calculated (Table 4) and listed in Table 7. As expected, the impedance mismatch between aerogels and tissue is very high (>90%) and contributes to high attenuation with strong posterior shadowing in aerogels.

## 5. Conclusions

Aerogels with physical properties spanning a wide range (0.32 < Y < 11.4 MPa, 0.04 < φ < 5 µm) have been successfully imaged in different physiologically-relevant environments using a portable diagnostic-grade US unit. For this, we developed a methodology to characterize and subsequently classify aerogels based on their acoustic properties. This methodology can be adopted industrially and can potentially streamline locating and tracking aerogel implants, as well as correctly interpreting US images from systems that contain aerogel implants.

The attenuation coefficient is an important marker of penetration depth and was an integral part of this investigation. The attenuation coefficient was found to increase with increasing frequency and did not show any dependency on the temperature in the range that was studied. The attenuation coefficient was also compared to the mechanical and structural properties of the aerogel samples (Y and φ). From these analyses and comparisons, it can be concluded that aerogels with a high Young’s modulus also have high attenuation (X-silica and X-silica-La_2_O_2_S:Eu aerogels), but aerogels with large pore diameter had the least attenuation (ARF-CA). Similarly, the impedance mismatch was calculated, and it was found that aerogels with high impedance mismatch have high echogenicity (hyperechoic), showing the US images with higher contrast between aerogel implants and their surroundings.

## Figures and Tables

**Table 1:** Aerogel types used in this study.

Aerogel Type	Material Type	References	Symbols
1. Crosslinked Silica	Polyurea-Crosslinked Silica Aerogel (X-silica aerogels)	[14,15,16,17,18,19,29,30]	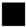
2. Phosphor doped X-silica	X-silica-La_2_O_2_S:Eu 10% doped	[36,37]	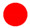
3. Shape Memory Polyurethane (SMPU)	SMPU Mix-14 TEG 1 mol/mol	[31,38,39]	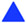
	SMPU Mix-18 DEG 0.25 mol/mol, TEG 0.5 mol/mol, TTEG 0.25 mol/mol		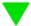
4. Carbon Aerogel (CA)	Acid-catalyzed Resorcinol Formaldehyde CA (ARF-CA)	[32,33]	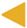
	Base-catalyzed Resorcinol Formaldehyde CA (BRF-CA)		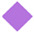
5. Polyurea-crosslinked calcium alginate (X-Ca-Alginate)	X-Ca-Alg-1	[34,35]	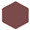
	X-Ca-Alg-2		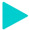

**Table 2:** Table summarizing equations used in this study.

Equation	Parameters		References
Y = Stress/Strain	(1)	Young’s modulus (Y):	[40]
Z = ρ √Y/ρ = ρv	(2a)	Acoustic Impedance (Z):	
v = √Y/ρ	(2b)	Speed of sound (v):	
I(t) = I_o_ exp(−αt)	(3a)	Change in US intensity:	
α = α_ο_f^n^	(3b)	Attenuation (α):	[41]
		Echogenicity (E):	
ΔE = [(MPI_ROI-3_ − MPI_ROI-1_)/MPI_ROI-1_] × 100	(4a)	At implant area:	
ΔE = [(MPI_ROI-4_ − MPI_ROI-2_)/MPI_ROI-2_] × 100	(4b)	At posterior shadowing:	
α = (γs^¤^)/2	(5a)	Attenuation (α):	[42]
γ = 4ϑ^#^/(πφ^2^)	(5b)	Pore-density (γ*):	
[(Z_Tissue_-Z_Aerogel_)/Z_Tissue_] × 100	(6)	Impedance mismatch:	

s^¤^ represents scattering cross section, ϑ^#^ represents porosity γ* pore density.

**Table 3:** Table showing the echogenicity classification used in this study with US images of aerogels inserted SC and SM being compared to US images of the tissue.

Change in Pixel Intensity in ROI (%)	Classification
~0%	Isoechoic
>1%	Hyperechoic
<−1%	Hypoechoic
~−100%	Anechoic

**Table 4:** Aerogel properties of each type used in this study: Young’s modulus (Y), Density (ρ), Speed (v), Acoustic Impedance (Z), and Pore Diameter (φ).

Aerogels	Y (Mpa)	Density ρ (kg/m^3^)	Speed v (m/s)	Z (Mrayl)	Pore Diameter φ (µm)
X-silica	8.35 ± 2.68	729.48	80.58	0.104	0.1 ± 0.012
X-silica-La_2_O_2_S:Eu	11.40 ± 2.20	939.20	90.21	0.126	0.13 ± 0.01
X-Ca-Alg-1	1.3 ± 0.2	88.76	48.80	0.026	0.19 ± 0.01
X-Ca-Alg-2	1.09 ± 0.02	150.49	42.48	0.026	0.18 ± 0.01
BRF-CA	0.95 ± 0.10	883.06	25.74	0.038	0.04 ± 0.001
ARF-CA	0.91 ± 0.09	826.62	28.83	0.040	5.00 ± 0.17
SMPU-Mix-18	0.40 ± 0.07	672.07	17.41	0.025	2.05 ± 0.19
SMPU-Mix-14	0.32 ± 0.07	637.01	14.75	0.022	2.67 ± 0.16

**Table 5:** US images and intensity maps of each aerogel type used in this study at a scan frequency of 6.5 MHz.

	Aerogel Type	US Image	Normalized Intensity Map	3D Intensity Map
1	Muscle	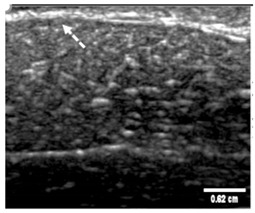	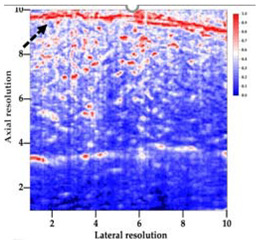	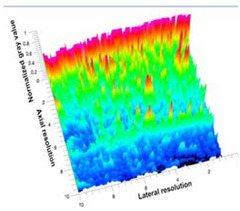
2	X-silica	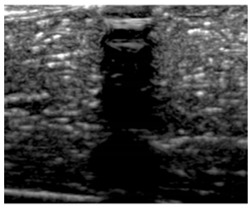	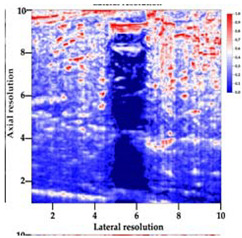	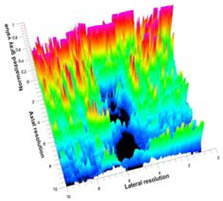
3	X-silica-La2O2S:Eu	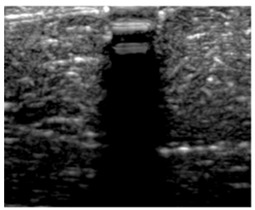	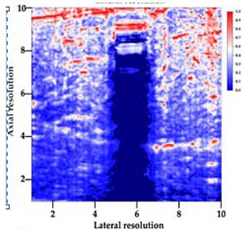	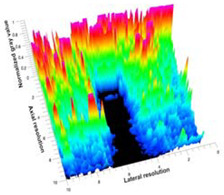
4	SMPU-Mix-14	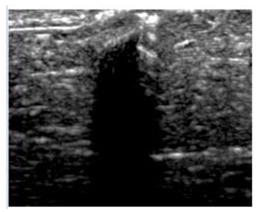	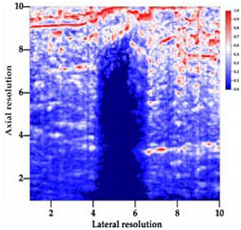	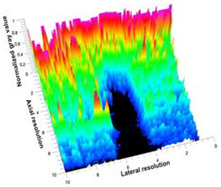
5	SMPU-Mix-18	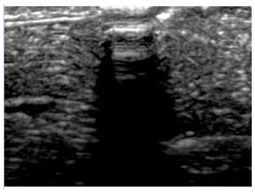	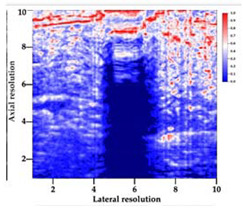	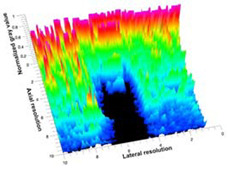
6	BRF-CA	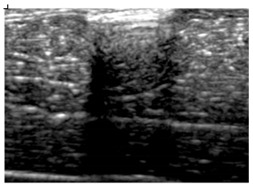	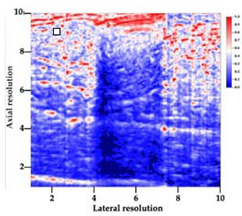	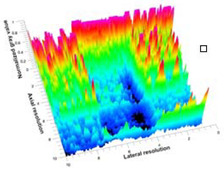
7	ARF-CA	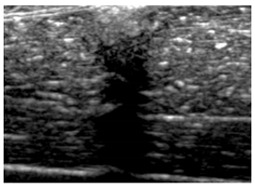	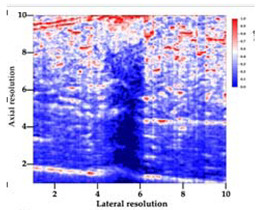	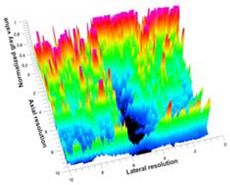
8	X-Ca-Alg-2	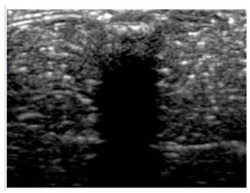	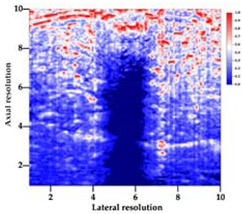	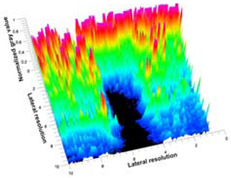
9	X-Ca-Alg-1	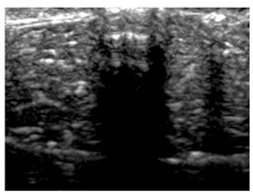	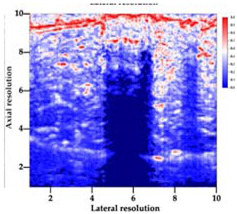	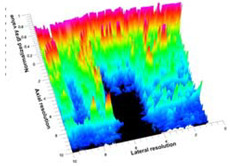

**Table 6:** Measurement of α for three different environments; Aq, SC and SM at a scan frequency of 8.5 MHz.

Aerogel	Aq Attenuation α (dB/cm)	SM Attenuation α (dB/cm)	SC Attenuation α (dB/cm)
X-silica-La_2_O_2_S:Eu	8.21 ± 0.14	13.76 ± 1.15	20.84 ± 0.14
X-silica	7.13 ± 0.15	9.80 ± 0.38	14.24 ± 0.98
SMPU-Mix-18	6.53 ± 0.01	9.12 ± 0.17	13.80 ± 0.07
SMPU-Mix-14	4.30 ± 0.88	5.96 ± 0.55	8.06 ± 0.70
X-Ca-Alg-2	3.54 ± 0.09	7.46 ± 0.39	10.54 ± 0.08
X-Ca-Alg-1	3.11 ± 0.12	7.42 ± 0.21	9.41 ± 0.35
BRF-CA	2.68 ± 0.35	6.60 ± 0.67	4.61 ± 0.16
ARF-CA	2.39 ± 0.44	4.77 ± 0.46	3.27 ± 0.05

**Table 7:** Aerogel imaging classifications and image characteristics based upon the optimal frequency, echogenicity.

Aerogel Type	Imaging Frequency (MHz)	Optimal Frequency (MHz)	Echogenicity		Image Characteristics	Impedance Mismatch (%)
			Subcutaneous	Submuscular		
X-silica	6.5–13.4	13.4 MHz	Hypoechoic	Least Hyperechoic	Distinct linear boundary	92.03
X-silica-La_2_O_2_S:Eu	6.5–13.4	13.4 MHz	Hypoechoic	Least Hyperechoic	Distinct linear boundary	90.28
SMPU-Mix-14	6.5–13.4	11 MHz	Hyperechoic	Moderately Hyperechoic	Irregular boundary	98.33
SMPU-Mix-18	6.5–13.4	11 MHz	Hyperechoic	Moderately Hyperechoic	Irregular boundary	98.23
BRF-CA	6.5–13.4	13.4 MHz	Hyperechoic	Hyperechoic	Waterfall appearance	97.16
ARF-CA	6.5–13.4	13.4 MHz	Hyperechoic	Hyperechoic	Waterfall appearance	97.12
X-Ca-Alg-2	6.5–13.4	11 MHz	Isoechoic	Strongly Hyperechoic	Irregular boundary	98.03
X-Ca-Alg-1	6.5–13.4	11 MHz	Isoechoic	Strongly Hyperechoic	Irregular boundary	97.97

## Data Availability

The data presented in this study are available upon request from the corresponding author.
